# Phase I Clinical Study of the Subunit Betulin-Adjuvanted Tetravalent Candidate Influenza Vaccine TetraFluBet

**DOI:** 10.3390/vaccines12091017

**Published:** 2024-09-05

**Authors:** Igor Krasilnikov, Irina Tcymbarevich, Anna Krasheninnikova, Maria Sukhova, Aleksandr Ivanov, Marina Stukova, Ekaterina Romanovskaya-Romanko, Tatiana Zubkova, Aleksandr Mironov, Dmitriy Lioznov

**Affiliations:** 1 CJSC Biotechnology Developments, 119136 Moscow, Russia; irina.tcymbarevich@hsci.ru (I.T.);; 2 Smorodintsev Research Institute of Influenza of the Ministry of Health of the Russian Federation, 197376 Saint Petersburg, Russiatanya_l@bk.ru (T.Z.);; 3 CRO “CTR-Pharma”, 119311 Moscow, Russia

**Keywords:** influenza vaccine, seasonal influenza, pandemic influenza, adjuvant, subunit vaccine, betulin

## Abstract

Objectives: This study aimed to determine the safety, tolerability and immunogenicity of TetraFluBet, an inactivated subunit influenza vaccine that contains a corpuscular immuno-adjuvant derived from natural betulin. Methods: We conducted a prospective, randomized, open-labeled, single-center, phase I trial. The study was conducted in two stages: 5 volunteers in stage I and 25 volunteers in stage II. Eligible participants received one single dose (20 μg/0.5 mL) of TetraFluBet intramuscularly. Participants were followed for adverse events and reactogenicity. Seroconversion rate, seroprotection level, geometric mean titers (GMTs) of virus-neutralizing antibodies, IFN-γ induction and cell-mediated immunity were assessed. Results: A total of 30 participants were enrolled. No vaccine-related serious adverse events were observed. The proportions of study participants with 4-fold seroconversions assessed by the HI assay (with 95% CIs) were 80.0% (62.7; 90.5), 70.0% (52.1; 83.3), 63.3% (45.5; 78.1) and 73.3% (55.6; 85.8) for influenza virus subtypes A (H1N1), A (H3N2), B1 and B2, respectively. Seroprotection levels (with 95% CIs) were 83.3% (66.4; 92.7), 83.3% (66.4; 92.7), 73.3% (55.6; 85.8) and 66.7% (48.8; 80.8), respectively. The fold increases in the GMTs of virus-neutralizing antibodies for subtype H1N1 was 6.50, for subtype H3N2 was 3.03, for subtype B1 was 3.56 and for subtype B2 was 6.07. The population of cytotoxic T-cells increased significantly in the post-vaccination period, indicating a strong CD3^+^CD8^+^ response. Conclusions: TetraFluBet tetravalent inactivated subunit vaccine with adjuvant demonstrated pronounced immunogenic properties, leading to the formation of both specific humoral and cellular immunity at a 20 μg dose.

## 1. Introduction

Among all respiratory viral diseases, influenza is the most severe and causes the most damage to public health and the economy. Due to the wide circulation of influenza viruses, there is a constant risk of seasonal epidemics and pandemics. Periodically, new pandemic strains emerge to which there is no population immunity, making the flu a particularly dangerous infection. According to the World Health Organization (WHO), during seasonal epidemics, up to 20% of the world’s population falls ill with influenza every year. This includes 5–10% of adults and 20–30% of children [1]. Complications of influenza, primarily pneumonia, determine the severity of the course and the fatal outcome. Severe forms are observed in 3–5 million cases, with deaths ranging from 250,000 to 500,000. The highest mortality rate is recorded among certain population groups: children, the elderly, pregnant women and people with weakened immune systems [2]. 

It is acknowledged that vaccination is a cost-effective measure in the prevention of influenza and acute respiratory viral infections. The inclusion of influenza vaccination in the National Immunization Schedule in 2006 and the ongoing implementation of a strategy to increase vaccination rates among target groups have clearly demonstrated the effectiveness of this approach. According to data from the Federal Service for Surveillance on Consumer Rights Protection and Human Wellbeing, consistent increases in vaccination rates across the country have led to a significant reduction in the number of influenza and severe acute respiratory syndrome (SARS-CoV-2) cases in the Russian Federation [3]. According to the Research Institute of Influenza, a part of the Ministry of Healthcare of the Russian Federation, the epidemic threshold for influenza morbidity in the Russian Federation was slightly exceeded in January 2018 by 20.6%, although there was no clear positive trend by the end of February and beginning of March 2018 [4]. Additionally, a recent study from Brazil has shown that people who have been vaccinated against influenza are partially protected from some of the more severe effects of coronavirus and are less likely to require emergency medical attention. An analysis of data from approximately 75,000 SARS-CoV-2 patients revealed a significant decrease in the incidences of strokes, deep vein thrombosis and sepsis, as well as a reduction in admissions to emergency rooms and intensive care units, among those who received the influenza vaccine. According to the study, individuals with SARS-CoV-2 who had not been vaccinated against influenza were 45–58% more likely to experience a stroke, approximately 40% were more likely to develop deep vein thrombosis and 36–45% were more likely to encounter sepsis. Additionally, they were more frequently admitted to the intensive care unit and emergency departments [5].

Influenza viruses that can infect humans are classified as RNA orthomyxoviruses and are divided into three main subtypes: A, B and C [6]. Most seasonal epidemics in humans are caused by influenza subtype A, while subtype B is more common among children and older adults, causing epidemics approximately every two to four years [7]. As these viruses undergo genetic mutation, there is often a change in their antigenic profile, leading to an “antigenic drift” that can cause annual outbreaks and epidemics [6].

In the 1980s, the influenza subtype B virus split into two distinct genetic groups, Yamagata and Victoria. Since 2001, these two strains have been circulating alternately or concurrently. This has significantly increased the difficulty in predicting which group will predominate during the upcoming influenza season [8]. At the same time, the WHO and public health authorities in various countries continue to recommend the use of trivalent influenza vaccines that only contain one subtype of influenza B. These vaccines do not always match the seasonal strain and, in some cases, both subtype B viruses circulate during the epidemic season.

During these periods, due to the lack of cross-reactivity with subtype B, the effectiveness of the trivalent vaccines is reduced [9]. Similar findings were reported in the United States in the 1999–2000 and 2012–2013 seasons when the B subtype vaccine provided only partial protection against the wild-type B strain in half of the cases [8]. A Cochrane meta-analysis of 38 systematic reviews of clinical trials published between 1966 and 2006, involving a total of 66,248 healthy adults aged 16–65 years, showed that the effectiveness of vaccination decreased when the vaccine strains did not match those circulating in the population during the season [9]. This fact is also supported by monitoring of the flu situation in Europe: the incidence of confirmed influenza subtype B cases ranged from 79% to 82% during the seasons between 1992 and 1993, and from 46% to 52% between 1998 and 1999 despite the gradual increase in the proportion of the vaccinated population (65 years and above) from 30% (1992–1993) to 70% (1998–1999) and then to 80% (2005–2006). [10]. In this context, ongoing efforts to improve vaccine formulations have become an essential part of the strategy to combat the spread of influenza.

The result of worldwide efforts in developments has been the emergence on the global market of tetravalent vaccines that contain both subtypes of influenza B virus (Yamagata and Victoria) [11,12,13,14,15,16,17]. According to estimates from the US Centers for Disease Control and Prevention, the use of a tetravalent flu vaccine could have reduced the incidence of flu in the 2007–2008 season by 1.3 million cases and decreased hospitalizations by 12,472 and deaths by 664 [8].

From the 2013–2014 season onwards, WHO recommended introducing a fourth component to the vaccine, an alternative strain, for countries in the northern hemisphere. A tetravalent vaccine was first approved for use in Europe in 2013. Currently, tetravalent inactivated vaccines, including Fluarix^®^ Tetra, Fluzone^®^ Quadrivalent, FluQuadri^®^, Afluria^®^ Quad and Flulaval^®^ Quadrivalent are available in Europe, the US, Canada, Australia and other parts of the world.

The present clinical trial is being conducted to evaluate the safety, tolerability and immunogenicity of TetraFluBet, a seasonal tetravalent influenza vaccine, in healthy adults aged 18 to 60 years old. This vaccine has undergone a comprehensive range of pre-clinical studies and has demonstrated a favorable safety profile [18]. Therefore, it has been approved for use in research on healthy volunteers. TetraFluBet is a tetravalent, inactivated, subunit influenza vaccine that contains a corpuscular immuno-adjuvant derived from natural betulin. This adjuvant (“Betusphere”) forms nanoparticles (NPs) that exhibit a spherical morphology analogous to that of viruses, thus facilitating their uptake by antigen-presenting cells. These NPs have a wide spectrum of immunopharmacological effects and enhance the immunogenicity and antigen stability of the vaccine [19,20]. Additionally, the adjuvant helps to increase immunologic memory and significantly reduces the antigen inoculation dose. Therefore, the total amount of hemagglutinin in one dose of TetraFluBet is 20 micrograms, which is half the amount typically found in trivalent, inactivated influenza vaccines without an adjuvant [21]. The results of the assessment of the safety, tolerability and immunogenicity of the TetraFluBet vaccine allow us to recommend further clinical trials of phases 2 and 3.

## 2. Materials and Methods

### 2.1. Study Objectives

This was a prospective, randomized, open-labeled, single-center phase 1 study to evaluate the safety, tolerability—including reactogenicity—and immunogenicity of a seasonal tetravalent influenza vaccine, TetraFluBet, in healthy participants aged 18–60 years.

### 2.2. Study Design Overview

Enrolled volunteers made several visits for surveillance to the research center during the study. The study was conducted in stages.

Stage I. The volunteers aged 18 and above who passed screening were assigned to Stage I (*n* = 5). The volunteers received one single dose (0.5 mL) intramuscularly. Safety, tolerability and reactogenicity of the TetraFluBet vaccine were assessed during the first 7 days after vaccination. Based on these results, a “Safety, tolerability and reactogenicity report” was generated by the investigator for approval by the Independent Data Monitoring Committee (IDMC), the Sponsor and the Local Ethics Committee (LEC). After review and approval by the IDMC, Sponsor and LEC, the study received permission to proceed from Stage I to Stage II.

Stage II. The volunteers who passed screening were assigned to Stage II (*n* = 25). The volunteers received one single dose (0.5 mL) intramuscularly. Eventually, a group comprising 30 volunteers took part in the phase 1 clinical trial and were administered the seasonal influenza vaccine, TetraFluBet, at 0.5 mL/dose.

**Figure 1:**
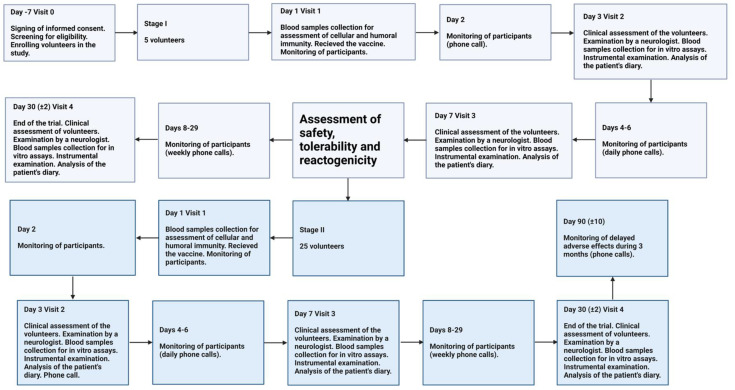
Study profile of the Phase 1 Trial.

The trial design is summarized in Figure 1.

### 2.3. Study Subjects

The study recruited female (*n* = 21) and male (*n* = 9) volunteers aged 18 to 60 years. The average age of the participants at the enrollment step was 35.57 ± 11.82 years. Before being enrolled in the study, all volunteers were provided with oral information and written materials about the tasks and methods of conducting the study and about the expected benefits and possible risks associated with participating in the study. They were instructed on the correct registration in the patient’s diary of all changes in well-being, body temperature, etc. Prior to enrollment and any manipulations, each participant was required to sign a written informed consent. The volunteers in all groups recruited for the study met the enrollment criteria defined in the protocol. The trial was conducted in accordance with the Declaration of Helsinki, the GCP rules of the Russian Federation and the European GCP Directive.

### 2.4. Formulations and Dosages of the Test Drug

One dose (0.5 mL) of the TetraFluBet vaccine contains 200 µg of natural betulin-based corpuscular adjuvant, 5 µg of influenza A (H1N1) virus, 5 µg of influenza A (H3N2) virus, 5 µg of influenza B (Yamagata-like) virus and 5 µg of influenza B (Victoria-like) virus in PBS buffer solution, pH 7.3, in 0.5 mL total volume. Subtypes of influenza viruses were chosen according to the WHO-recommended composition of influenza virus vaccines for use in the 2018–2019 northern hemisphere influenza season for quadrivalent vaccines. The technology used for TetraFluBet manufacturing was described previously [22].

### 2.5. Safety and Immunogenicity Outcomes

#### 2.5.1. Study Endpoints

The endpoints for this study were safety and immunogenicity. The primary safety endpoints were adverse event (AE) or serious adverse event (SAE) information collected in the clinic and via memory aids/diary cards, concomitant medications and periodic targeted physical examination assessments. Immunogenicity endpoints were vaccine-induced immunity.

Safety Outcomes:

The severity of the AE was determined according to the following classification:

1. Mild effects are described as having minor significance and being easily tolerated by the subject, causing minimal inconvenience and not interfering with daily activities.
2. Moderate effects cause discomfort and interference with daily activities but are not life-threatening.
3. Severe effects are those that may be life-threatening and interfere significantly with normal daily life.

#### 2.5.2. Assessment Criteria for Safety, Tolerability and Reactogenicity

1. The incidence rates and severity grades of local and systemic adverse reactions seven days after vaccination, according to subjective (patient’s diary) and objective (medical examination) data.
2. Incidence rates and severity of AEs/SAEs.
3. Proportion of participants who used antipyretics during the study, as well as the duration of their use.
4. Frequency of clinically significant deviations from reference values in the laboratory, instrumental studies, vital signs and physical examination.

#### 2.5.3. Assessment Criteria for Immunogenicity

Seroconversion rate

The seroconversion rate was determined by fold-increases in the GMTs of hemagglutinating antibodies to influenza virus subtypes A (H1N1), A (H3N2), B1 and B2 30 days after vaccination compared with the initial levels. Titers of specific antibodies were determined using the HI assay at Visits 1 (before vaccination) and 4 (30 days later after vaccination). 

The proportion of participants with more than a 4-fold increase in GMTs 30 ± 2 days after vaccination considering the initial level of antibodies to influenza virus subtypes included in the vaccine is indicated as a fold increase in the text. Individuals with GMTs ˃1:20 and ≤1:20 were analyzed separately and together.

Seroprotection level

Assessment of the proportion of individuals in whom the titer of hemagglutinating antibodies was >1:40 30 ± 2 days after vaccination was also carried out considering the initial level of antibodies to influenza virus subtypes included in the vaccine.

GMTs of virus-neutralizing antibodies before and 30 ± 2 days after vaccination

The level of virus-neutralizing antibodies in the sera of individuals vaccinated with seasonal influenza TetraFluBet vaccine was calculated using logarithmic data transformation using the same approach as for HI assay data.

Cell immunity Assessment

Cellular immunity after a single vaccination of the participants with the seasonal influenza tetravalent TetraFluBet vaccine was assessed by determining the level of CD3^+^CD4^+^ (T-helper) and CD3^+^CD8^+^ (T-cytotoxic) cell populations before and 30 ± 2 days after vaccination.

IFN-γ Assessment

IFN-γ induction was assessed at Visits 1 (before vaccination) and 4 (30 days after vaccination). The study participants were analyzed depending on the initial levels of antibodies.

### 2.6. Statistical Analysis

Demographic data and initial clinical and laboratory characteristics were evaluated in all volunteers included in the study. The estimated parameters are presented depending on the type of data distribution using descriptive statistics (mean, standard deviation, standard error of the mean, lower and upper bounds of 95% CI, minimum, maximum). The choice of parametric or nonparametric criteria for data presentation was determined by the results of a distribution analysis based on data evaluation using the Shapiro–Wilk criterion (conclusion on the presence/absence of statistically significant differences in the distribution of the corresponding indicator from the law of normal distribution). Throughout this article, asterisks (*) denote significant differences at *p* < 0.05.

## 3. Results and Discussion

### 3.1. Vaccine Safety

The analysis of data on safety, tolerability and reactogenicity was carried out in the TS (treatment success) population. The population for the assessment of safety, tolerability and reactogenicity consisted of 30 volunteers who received the TetraFluBet vaccine. TS, ITT (intention-to-treat) and PP (per protocol) groups were matched in this study. All 30 participants included in the study met the criteria for inclusion based on anthropometric measurements, vital signs, physical examinations and a neurological examination. There were no clinically significant deviations in laboratory parameters that required further evaluation or could have negatively impacted the safety of the volunteer’s participation in the study. No clinically significant deviations were reported during electrocardiograms (ECGs) or external respiratory tests.

During the 7-day follow-up period after administration of the drug, 11 (36.67%) AEs occurred in eight (26.67%) of the volunteers (Table 1). AEs related to vaccination were determined in six patients, with four local reactions recorded in four volunteers (#05, #10, #12, #32), resulting in an incidence rate of 13.33%. All four reactions were associated with pain at the injection site, with a proportion of 10% of the volunteers experiencing local reactions. Registered reactions were mild in 100% of the cases, with no severe reactions reported. In addition, five systemic reactions were reported in four other volunteers (#07, #10, #12, #24), with an incidence rate for systemic reactions of 16.66%, of which three (#07, #10, #24) were associated with fever and two (#10, #12) were associated with discomfort. The proportion of those experiencing systemic reactions was also 13% (four volunteers). Of the systemic reactions recorded, 80% (four AEs) were mild and 20% (one AE) moderate.

A relationship between vaccination and the reported symptoms was not established in 6.67% of the patients (#9, #22); however, both symptoms were mild, representing acute respiratory viral infections in 3.33% (#9) of cases and cough in 3.33% (#22). All reported events resolved with full recovery and did not require any further treatment. No SAEs were reported during the study period. No cases of the use of antipyretics by volunteers during the surveillance period were recorded; therefore, during the study, 0% of participants took antipyretic medications, and the duration of use of these medications was 0 days. No clinically significant deviations from reference laboratory values were observed during the study. Clinically insignificant deviations from normal ranges were minor, had no clinical significance and were attributed by the researchers to physiological factors such as the body’s response to vaccine administration, the phase of the menstrual cycle and dietary habits that are not related to pathology and do not pose a risk of developing pathology if these volunteers continue to participate in the research. These factors were not exclusion criteria in accordance with the trial protocol and did not indicate the presence of a pathological process within the body.

Based on the results of monitoring the main vital parameters, no negative trends in the health condition of the volunteers were observed during the study period (Tables S1 and S2). Furthermore, there were no abnormalities in the evaluation of neurological status or according to instrumental examinations. During the study, one clinically relevant AE was identified in one volunteer (3.33%), which was mild in severity and led to complete recovery without the need for corrective treatment (volunteer #9). No association with the vaccination was established. According to medical examination results, no other changes in the volunteers’ health status were detected. Thus, based on the results of the study conducted, the safety, good tolerability and low reactogenicity of the new TetraFluBet tetravalent inactivated subunit adjuvanted influenza vaccine were confirmed.

### 3.2. Vaccine Immunogenicity

**Figure 2:**
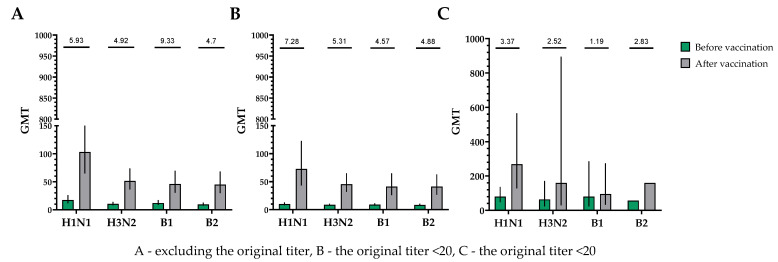
GMTs of hemagglutinating antibodies to H1N1, H3N2, B1 and B2 subtypes before and after vaccination. (**A**) GMTs of hemagglutinating antibodies to H1N1, H3N2, B1 and B2 subtypes for all the participants (*n* = 30). GMTs (95% CIs) before and after vaccination. (**B**) GMTs (95% CIs) of hemagglutinating antibodies to H1N1, H3N2, B1 and B2 subtypes for participants with initial titers ≤ 1:20. GMTs (95% CIs) before and after vaccination. (**C**) GMTs of hemagglutinating antibodies to H1N1, H3N2, B1 and B2 subtypes for participants with initial titers > 1:20. GMTs (95% CIs) before and after vaccination. Numbers on the graph indicate the fold increases. Error bars indicate 95% CIs. Raw numbers are available in Table S3.

The population for evaluating efficacy (immunogenicity) consisted of 30 vaccinated participants who completed the study in accordance with the protocol (PP and TS populations). A single immunization of all study participants, without taking into account the initial values of the titers of hemagglutinating antibodies, provided the following indicators of immunogenicity in HI assays for influenza viruses of subtypes A (H1N1), A (H3N2), B1 and B2 (Figure 2).

**Figure 3:**
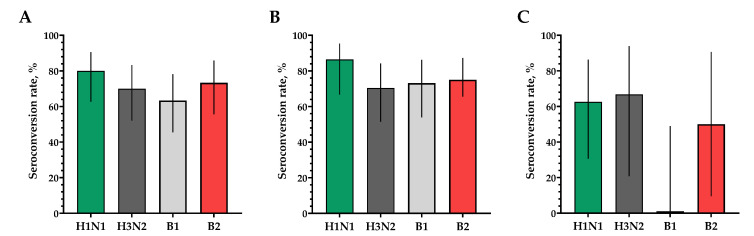
The proportion of study participants with >4-fold seroconversion rates of hemagglutinating antibodies to H1N1, H3N2, B1 and B2 subtypes. (**A**) Seroconversion rates (with 95% CIs) of hemagglutinating antibodies to H1N1, H3N2, B1 and B2 subtypes for all the participants (*n* = 30). (**B**) Seroconversion rates (with 95% CIs) of hemagglutinating antibodies to H1N1, H3N2, B1 and B2 subtypes for participants with initial titers ≤ 1:20. (**C**) Seroconversion rates (with 95% CIs) of hemagglutinating antibodies to H1N1, H3N2, B1 and B2 subtypes for participants with initial titers > 1:20. Error bars indicate 95% CIs. Raw numbers are available in Table S3.

**Figure 4:**
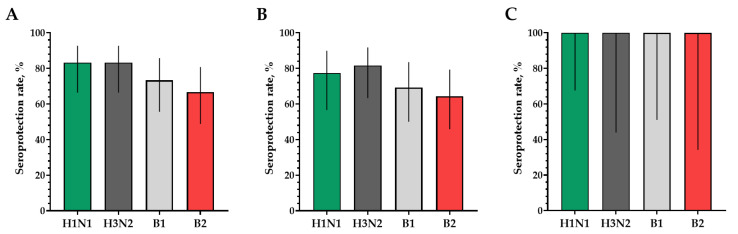
Seroprotection rates showing GMTs of hemagglutinating antibodies to H1N1, H3N2, B1 and B2 subtypes for participants with post-vaccination protective titers > 1:40. (**A**) GMTs of hemagglutinating antibodies to H1N1, H3N2, B1 and B2 subtypes for all the participants (*n* = 30). (**B**) GMTs of hemagglutinating antibodies to H1N1, H3N2, B1 and B2 subtypes for participants with initial titers ≤ 1:20. (**C**) GMTs of hemagglutinating antibodies to H1N1, H3N2, B1 and B2 subtypes for participants with initial titers > 1:20. Error bars indicate 95% CIs. Raw numbers are available in Table S3.

The proportions of study participants with 4-fold seroconversions, excluding those with an initial titer of antibodies seroconversion rates (95% CI)/fold increase was 80.0% (62.7; 90.5)/5.93 for H1N1, 70.0% (52.1; 83.3)/4.92 for H3N2, 63.3% (45.5; 78.1)/9.33 for B1 and 73.3% (55.6; 85.8)/4.70 for B2 (Figure 3). Seroprotection levels with 95% CIs were 83.3% (66.4; 92.7) for H1N1, 83.3% (66.4; 92.7) for H3N2, 73.3% (55.6; 85.8) for B1 and 66.7% (48.8; 80.8) for B2 (Figure 4).

In a population of volunteers with initial antibody titers <1:20 and >1:20, a significant increase in the titers of hemagglutinating antibodies in vaccinated study participants was also noted. In a population of volunteers with initial antibody titers < 1:20, seroconversion rates (fold increase) of 86.4% (7.28), 70.4% (5.31), 73.1% (4.57) and 75.0% (4.88) were observed for H1N1, H3N2, B1 and B2 subtypes, respectively (Figure 3). In a population of volunteers with initial antibody titers > 1:20, seroconversion rates (fold increase) of 62.5% (3.37), 66.7% (2.52), 0% (1.19) and 50% (2.83) were observed. Seroprotection levels were 100.0%, 100.0%, 100.0% and 100.0% for H1N1, H3N2, B1 and B2 subtypes, respectively (Figure 4).

**Figure 5:**
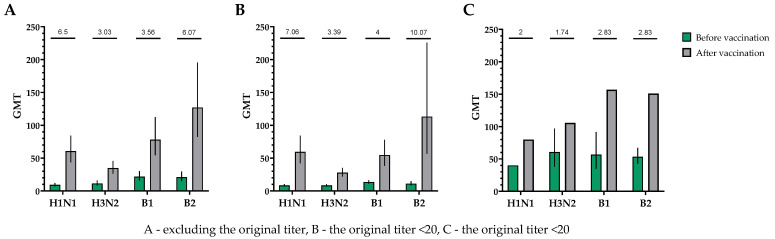
GMTs of neutralizing antibodies to H1N1, H3N2, B1 and B2 subtypes before and after vaccination. (**A**) GMTs (with 95% CIs) of neutralizing antibodies to H1N1, H3N2, B1 and B2 subtypes for all the participants (*n* = 30). (**B**) GMTs (with 95% CIs) of neutralizing antibodies to H1N1, H3N2, B1 and B2 subtypes for participants with initial titers ≤ 1:20. (**C**) GMTs (with 95% CIs) of neutralizing antibodies to H1N1, H3N2, B1 and B2 subtypes for participants with initial titers > 1:20. Numbers on the graph indicate the fold increase. Error bars indicate 95% CIs. Raw numbers are available in Table S3.

When investigating the humoral immune response in neutralization reactions, a significant increase in virus-neutralizing antibodies was observed in the blood serum of study participants after a single intramuscular immunization with TetraFluBet, a tetravalent inactivated subunit adjuvanted influenza vaccine. In the population, regardless of the initial level of virus-neutralizing antibodies, the fold increase in the antibody titer for subtype H1N1 was 6.50, for serotype H3N2 was 3.03, for serotype B1 was 3.56 and for serotype B2 was 6.07 (Figure 5).

**Figure 6:**
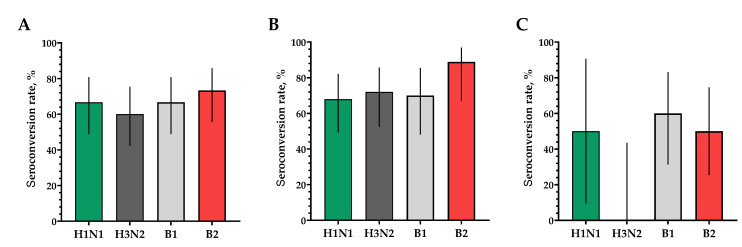
The proportions of study participants with >4-fold seroconversion rate of neutralizing antibodies to H1N1, H3N2, B1 and B2 subtypes 30 days after vaccination. (**A**) Seroconversion rates (with 95% CIs) of neutralizing antibodies to H1N1, H3N2, B1 and B2 subtypes for all the participants (*n* = 30). (**B**) Seroconversion rates (with 95% CIs) of neutralizing antibodies to H1N1, H3N2, B1 and B2 subtypes for participants with initial titers ≤ 1:20. (**C**) Seroconversion rates (with 95% CIs) of neutralizing antibodies to H1N1, H3N2, B1 and B2 subtypes for participants with initial titers > 1:20. Error bars indicate 95% CIs. Raw numbers are available in Table S3.

**Figure 7:**
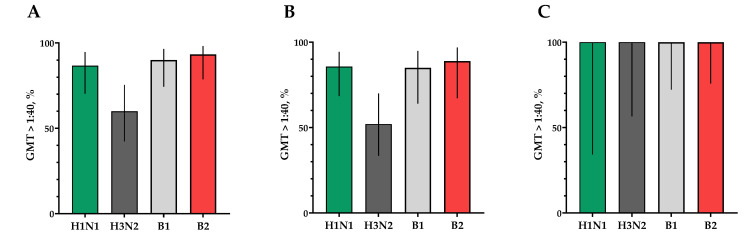
Seroprotection rates showing GMTs of neutralizing antibodies to H1N1, H3N2, B1 and B2 subtypes for participants with post-vaccination protective titers > 1:40. (**A**) GMTs (with 95% CIs) of neutralizing antibodies to H1N1, H3N2, B1 and B2 subtypes for all the participants (*n* = 30). (**B**) GMTs (with 95% CIs) of neutralizing antibodies to H1N1, H3N2, B1 and B2 subtypes for participants with initial titers ≤ 1:20. (**C**) GMTs (with 95% CIs) of neutralizing antibodies to H1N1, H3N2, B1 and B2 subtypes for participants with initial titers > 1:20. Error bars indicate 95% CIs. Raw numbers are available in Table S3.

The proportion of study participants with 4-fold seroconversions on the 30th day after vaccination to serotype H1N1 was 66.7%, to serotype H3N2 was 60%, to serotype B1 was 66.7% and to serotype B2 was 73.3% (Figure 6A). The number of persons (95% CIs) with conditionally protective antibody ranges above 1:40 to the H1N1 serotype was 86.7% (70.3; 94.7), to the H3N2 serotype was 60% (42.3; 75.4), to the B1 serotype was 90.0% (74.4; 96.5) and to the B2 serotype was 93.3% (78.7; 98.2) (Figure 7A).

In a population of volunteers with initial antibody titers <1:20 and >1:20, a significant increase in titers of virus-neutralizing antibodies was also noted in vaccinated study participants, which indicates the potential high efficacy of the vaccine (Figure 6B,C and Figure 7B,C).

**Figure 8:**
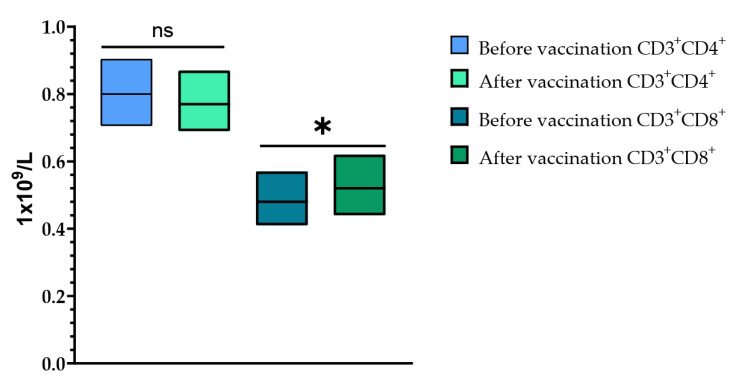
CD3^+^CD4^+^ and CD3^+^CD8^+^ cell populations before and after vaccination. The post-vaccination cell-mediated immune response demonstrated that the absolute numbers (1 × 10^9^/L) of CD4^+^ T-cells did not change, while the CD8^+^ population increased significantly. CD3^+^CD4^+^ (1 × 10^9^/L (with 95% CIs) before vaccination was 0.80 (0.71; 0.90) and after vaccination was 0.77 (0.69; 0.87); *p* > 0.05 (*p* = 0.33799). CD3^+^CD8^+^ (1 × 10^9^/L (with 95% CIs) before vaccination was 0.48 (0.41; 0.57) and after vaccination was 0.52 (0.44; 0.62) (*p* = 0.03165). Error bars indicate 95% CIs; *p* ≤ 0.05 is indicated with *.

Cell immunity was assessed after a single vaccination with TetraFluBet vaccine by determining the levels of CD3^+^CD4^+^ (T-helper cells) and CD3^+^CD8^+^ (T-cytotoxic cells) at Visits 1 (before vaccination) and 4 (30 ± 2 days after vaccination) (Figure 8). When assessing the levels of CD3^+^CD4^+^ (T-helper cells) before vaccination and 30 ± 2 days after vaccination, it was shown that a single vaccination with TetraFluBet influenza vaccine did not lead to a statistically significant difference in values both in fold increase and percentage (*p* > 0.05). Evaluation of CD3^+^CD8^+^ (T-cytotoxic cells) indicated a statistically significant increase in absolute values (*p* = 0.03165) after vaccination, which indicates the stimulating effect of the seasonal influenza tetravalent TetraFluBet vaccine on the cellular part of the immune response. We speculate that CD8^+^ T-cell responses may also contribute to protection against influenza.

**Figure 9:**
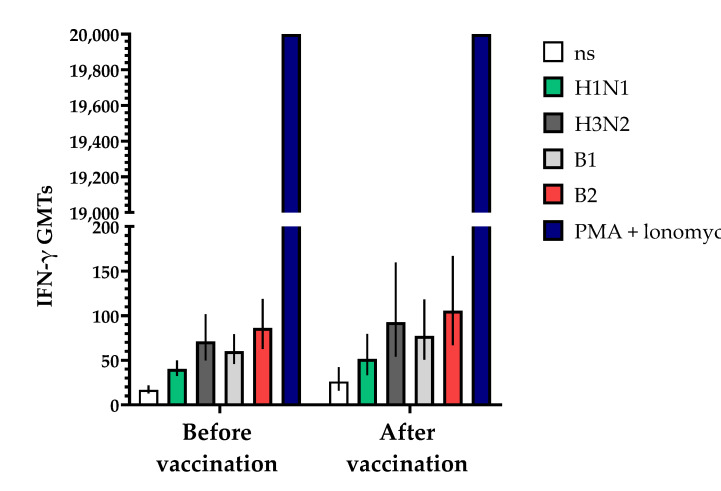
Effects of TetraFluBet on induction of IFN-γ 30 ± 2 days after vaccination. IFN-γ GMT levels (with 95% CIs) before and after vaccination show a trend (non-significant) toward an increase. Ns—non stimulated; stimulated by specific antigens (H1N1, H3N3, B1, B2); PMA (phorbol 12-myristate 13-acetate) + ionomycin—positive control. Error bars indicate 95% CIs. Raw numbers are available in Table S3.

The study of cell immunity also involved an assessment of the induction of interferon-gamma (IFN-γ) before and 30 ± 2 days after vaccination with the vaccine (Figure 9). The studies showed that the geometric mean titers (GMTs) of IFN-γ following a single vaccination increased in all the blood serum samples from vaccinated volunteers that were studied. The fold increase in IFN-γ levels in unstimulated blood samples increased by 1.56-fold compared to the initial data, while in samples stimulated with specific antigens of influenza virus serotypes H1N1, B1, H3N2 and B2, the fold increases (with 95% CIs) were 1.28 (0.91; 2.69), 1.28 (0.84; 1.94), 1.30 (0.89; 1.90) and 1.22 (0.86; 1.74), respectively, with a positive control of phorbol 12-myristate 13-acetate (PMA) + ionomycin > 20,000 being used.

In summary, we demonstrated that the TetraFluBet vaccine candidate induces hemagglutination-inhibiting and virus-neutralizing antibodies. It also led to a statistically significant increase in the number of CD3^+^CD8^+^ cells (T-cytotoxic lymphocytes) and a trend towards an increase in the levels of interferon-gamma (IFN-γ) for all four strains of influenza virus included in the formulation. The induction of both specific humoral and cellular immunity indicates its potential to be effective against seasonal influenza infections.

## 4. Conclusions

The data from the present study indicate that Tetra FluBet, a tetravalent inactivated subunit influenza vaccine with a corpuscular immuno-adjuvant derived from natural betulin, showed good immunogenicity. The safety, tolerability and low reactogenicity of the preparation were confirmed. Further immunogenicity and safety studies in larger placebo- or comparator-controlled clinical trials in phases 2 and 3 are needed to confirm the results from the phase 1 study, as well as to demonstrate efficacy. Additionally, further studies are needed for dose-finding and to demonstrate the added value of the novel betulin adjuvant (“Betusphere”).

## Figures and Tables

**Table 1:** The incidence rates of local and systemic adverse reactions/days after each vaccination in phase 1 clinical trial.

Organs	Description	Mild		Moderate		Severe		SAE		Total *n*/%		Sum *n*/%
		Related to Vaccination	N/Related to Vaccination	Related to Vaccination	N/Related to Vaccination	Related to Vaccination	N/Related to Vaccination	Related to Vaccination	N/Related to Vaccination	Related to Vaccination	N/Related to Vaccination	Related to Vaccination + N/Related to Vaccination
Local reactions	injection-site pain	4 (#05, #10, #12, #32)	0	0	0	0	0	0	0	4/13.33	0	4/13.33
Systemic reactions	fever	2 (#07, #24)	0	1 (#10)	0	0	0	0	0	3/10.00	0	3/10.00
	malaise	2 (#10, #12)	0	0	0	0	0	0	0	2/6.67	0	2/6.67
Respiratory system	acute viral infection	0	1 (#09)	0	0	0	0	0	0	0	1/3.33	1/3.33
	cough	0	1 (#22)	0	0	0	0	0	0	0	1/3.33	1/3.33

Group (*n* = 60 observations). Volunteer number is indicated in parenthesis (#). N/Related to vaccination—not related to vaccination.

## Data Availability

The datasets presented in this study are available from the corresponding author on a reasonable request.

## Supplementary Materials

The following supporting information can be downloaded at: https://www.mdpi.com/article/10.3390/vaccines12091017/s1, Table S1: Descriptive statistics of laboratory tests performed on Visit 2 and Visit 4; Table S2: Descriptive statistics of main vital parameters on Visits 1–4; Table S3: Data for Figure 2, Figure 3, Figure 4, Figure 5, Figure 6, Figure 7 and Figure 9.
