# Supplementary material for: Phase I Clinical Study of the Subunit Betulin-Adjuvanted Tetravalent Candidate Influenza Vaccine TetraFluBet
Source: Vaccines (Basel). 2024 Sep 5;12(9):1017. doi: 10.3390/vaccines12091017 (PMC11436025; doi:10.3390/vaccines12091017)
Supplement: Supplementary file 1 [file vaccines-12-01017-s001.zip › Supplement Tables S1-S2.docx]

**Supplement**

**Table S1**

***Descriptive statistics of laboratory tests performed on Visit 2 and Visit 4***

| **Parameter** | **Mean** | **SD** | **SEM** | **95 % CI**  **lower** | **95 % CI upper** | **Min** | **Max** |
| --- | --- | --- | --- | --- | --- | --- | --- |
| ***Visit 2*** | | | | | | | |
| *Full blood panel* | | | | | | | |
| Hematocrit, % | 39.53 | 3.03 | 0.55 | 38.4 | 40.66 | 33.8 | 46.2 |
| Hemoglobin, g/L | 133.73 | 12.51 | 2.28 | 129.06 | 138.4 | 117 | 162 |
| Red blood cells, 10^12^/L | 4.56 | 0.35 | 0.06 | 4.43 | 4.69 | 3.9 | 5.46 |
| Platelets, 10^9^/L | 265.13 | 65.66 | 11.99 | 240.62 | 289.65 | 155 | 458 |
| White blood cells, 10^9^/L | 5.96 | 1.22 | 0.22 | 5.5 | 6.42 | 3.5 | 8.82 |
| Segmented neutrophils, 10^9^/L | 3.23 | 0.96 | 0.17 | 2.87 | 3.59 | 1.22 | 5.82 |
| Segmented neutrophils, % | 54.4 | 7.78 | 1.42 | 51.5 | 57.3 | 35 | 68 |
| Eosinophils, 10^9^/L | 0.18 | 0.14 | 0.03 | 0.13 | 0.23 | 0 | 0.6 |
| Eosinophils, % | 3.03 | 2.04 | 0.37 | 2.27 | 3.8 | 0 | 9 |
| Basophils, 10^9^/L | 0.02 | 0.02 | 0 | 0.02 | 0.03 | 0 | 0.06 |
| Basophils, % | 0.57 | 0.5 | 0.09 | 0.38 | 0.75 | 0 | 1 |
| Monocytes, 10^9^/L | 0.53 | 0.14 | 0.02 | 0.48 | 0.58 | 0.33 | 0.79 |
| Monocytes, % | 9.07 | 2.08 | 0.38 | 8.29 | 9.84 | 6 | 14 |
| Lymphocytes, 10^9^/L | 1.88 | 0.52 | 0.09 | 1.69 | 2.07 | 0.87 | 3.01 |
| Lymphocytes, % | 31.77 | 7.65 | 1.4 | 28.91 | 34.63 | 19 | 46 |
| ESR (Erythrocyte sedimentation rate), mm/hour | 9.77 | 6.84 | 1.25 | 7.21 | 12.32 | 2 | 27 |
| *Biochemical blood test* | | | | | | | |
| AST (Aspartate transaminase), Units/L | 19.9 | 5.23 | 0.96 | 17.95 | 21.85 | 11 | 32 |
| ALT (Alanine aminotransferase), Units/L | 20.3 | 7.71 | 1.41 | 17.42 | 23.18 | 11 | 42 |
| Alkaline Phosphatase, U/L | 66.53 | 16.84 | 3.07 | 60.25 | 72.82 | 34 | 119 |
| GGT (Gamma-glutamyl transferase), U/L | 19.14 | 6.03 | 1.1 | 16.89 | 21.39 | 12.2 | 33.6 |
| Total Bilirubin, mmol/L | 7.82 | 2.72 | 0.5 | 6.81 | 8.83 | 4.3 | 13.9 |
| Total Protein, g/L | 71.8 | 3.16 | 0.58 | 70.62 | 72.98 | 67 | 79 |
| Glucose, mmol/L | 4.83 | 0.55 | 0.1 | 4.62 | 5.03 | 3.9 | 5.9 |
| Creatinine, mmol/L | 77.8 | 6.34 | 1.16 | 75.43 | 80.17 | 64 | 95 |
| Urea, mmol/L | 4.29 | 0.98 | 0.18 | 3.93 | 4.66 | 2.3 | 6.4 |
| *IgE* | | | | | | | |
| IgE, IU/mL | 32,53 | 27,46 | 5,01 | 22,28 | 42,79 | 2 | 101 |
| *Urine analysis* | | | | | | | |
| Specific gravity of urine, U | 1021,67 | 4,97 | 0,91 | 1019,81 | 1023,52 | 1010 | 1030 |
| pH | 5,97 | 0,64 | 0,12 | 5,73 | 6,21 | 5 | 7 |
| Protein | 0,01 | 0,03 | 0,01 | -0,01 | 0,02 | 0 | 0,15 |
| Glucose | 0 | 0 | 0 | 0 | 0 | 0 | 0 |
| Bilirubin | 0,29 | 1,57 | 0,29 | -0,3 | 0,87 | 0 | 8,6 |
| Urobilinogen | 0 | 0 | 0 | 0 | 0 | 0 | 0 |
| Ketone bodies | 0 | 0 | 0 | 0 | 0 | 0 | 0 |
| Nitrites | 0 | 0 | 0 | 0 | 0 | 0 | 0 |
| Squamous epithelium | 3,1 | 0,99 | 0,18 | 2,73 | 3,47 | 1 | 5 |
| Red blood cells | 2 | 10,95 | 2 | -2,09 | 6,09 | 0 | 60 |
| White blood cells | 2,83 | 1,12 | 0,2 | 2,42 | 3,25 | 1 | 5 |
| Cylinders | 0 | 0 | 0 | 0 | 0 | 0 | 0 |
| Crystals | 0 | 0 | 0 | 0 | 0 | 0 | 0 |
| Bacteria | 0 | 0 | 0 | 0 | 0 | 0 | 0 |
| ***Visit 4*** | | | | | | | |
| *Full blood panel* | | | | | | | |
| Hematocrit, % | 39.57 | 2.87 | 0.52 | 38.5 | 40.64 | 34.5 | 45.1 |
| Hemoglobin, g/L | 132.83 | 12.55 | 2.29 | 128.15 | 137.52 | 111 | 159 |
| Red blood cells, 10^12^/L | 4.57 | 0.34 | 0.06 | 4.45 | 4.7 | 3.84 | 5.24 |
| Platelets, 10^9^/L | 269.63 | 77.65 | 14.18 | 240.64 | 298.63 | 153 | 572 |
| White blood cells, 10^9^/L | 5.92 | 1.23 | 0.23 | 5.46 | 6.38 | 3.28 | 8.21 |
| Segmented neutrophils, 10^9^/L | 0.47 | 1.07 | 0.2 | 0.07 | 0.87 | 0 | 5 |
| Segmented neutrophils, % | 3.28 | 0.92 | 0.17 | 2.94 | 3.62 | 1.51 | 5.44 |
| Eosinophils, 10^9^/L | 54.67 | 7.4 | 1.35 | 51.9 | 57.43 | 43 | 75 |
| Eosinophils, % | 0.16 | 0.11 | 0.02 | 0.12 | 0.2 | 0.01 | 0.57 |
| Basophils, 10^9^/L | 2.83 | 1.62 | 0.3 | 2.23 | 3.44 | 0 | 7 |
| Basophils, % | 0.03 | 0.02 | 0 | 0.03 | 0.04 | 0.01 | 0.07 |
| Monocytes, 10^9^/L | 0.63 | 0.49 | 0.09 | 0.45 | 0.82 | 0 | 1 |
| Monocytes, % | 0.51 | 0.16 | 0.03 | 0.45 | 0.57 | 0.3 | 1.09 |
| Lymphocytes, 10^9^/L | 8.77 | 2.14 | 0.39 | 7.97 | 9.57 | 5 | 13 |
| Lymphocytes, % | 1.91 | 0.52 | 0.09 | 1.71 | 2.1 | 0.9 | 3.05 |
| ESR (Erythrocyte sedimentation rate), mm/hour | 32.63 | 7.18 | 1.31 | 29.95 | 35.31 | 12 | 48 |
| *Biochemical blood test* | | | | | | | |
| AST (Aspartate transaminase), Units/L | 19.1 | 4.6 | 0.84 | 17.38 | 20.82 | 12 | 28 |
| ALT (Alanine aminotransferase), Units/L | 20.33 | 6.16 | 1.12 | 18.03 | 22.63 | 10 | 36 |
| Alkaline Phosphatase, U/L | 67.33 | 17.49 | 3.19 | 60.8 | 73.86 | 38 | 112 |
| GGT (Gamma-glutamyl transferase), U/L | 21.48 | 6.83 | 1.25 | 18.93 | 24.03 | 11.2 | 39.3 |
| Total Bilirubin, mmol/L | 8.89 | 4.8 | 0.88 | 7.1 | 10.68 | 2.8 | 19.2 |
| Total Protein, g/L | 71.83 | 2.59 | 0.47 | 70.87 | 72.8 | 67 | 77 |
| Glucose, mmol/L | 5.25 | 0.52 | 0.1 | 5.05 | 5.45 | 4.2 | 5.9 |
| Creatinine, mmol/L | 77 | 7.57 | 1.38 | 74.17 | 79.83 | 64 | 93 |
| Urea, mmol/L | 4.17 | 1.13 | 0.21 | 3.75 | 4.6 | 2.3 | 6.9 |
| *IgE* | | | | | | | |
| IgE, IU/mL | 43.47 | 26.32 | 4.81 | 33.64 | 53.3 | 5 | 89 |
| *Urine analysis* | | | | | | | |
| Specific gravity of urine, U | 1024.67 | 5.24 | 0.96 | 1022.71 | 1026.62 | 1010 | 1030 |
| pH | 5.67 | 0.62 | 0.11 | 5.43 | 5.9 | 5 | 6.5 |
| Protein | 0 | 0 | 0 | 0 | 0 | 0 | 0 |
| Glucose | 0 | 0 | 0 | 0 | 0 | 0 | 0 |
| Bilirubin | 0 | 0 | 0 | 0 | 0 | 0 | 0 |
| Urobilinogen | 0 | 0 | 0 | 0 | 0 | 0 | 0 |
| Ketone bodies | 0 | 0 | 0 | 0 | 0 | 0 | 0 |
| Nitrites | 0 | 0 | 0 | 0 | 0 | 0 | 0 |
| Squamous epithelium | 3.3 | 1.06 | 0.19 | 2.91 | 3.69 | 2 | 5 |
| Red blood cells, 10^9^/L | 0.3 | 1.64 | 0.3 | -0.31 | 0.91 | 0 | 9 |
| White blood cells, 10^9^/L | 2.5 | 0.73 | 0.13 | 2.23 | 2.77 | 1 | 4 |
| Cylinders | 0 | 0 | 0 | 0 | 0 | 0 | 0 |
| Crystals | 0 | 0 | 0 | 0 | 0 | 0 | 0 |
| Bacteria | 0 | 0 | 0 | 0 | 0 | 0 | 0 |

**Table S2**

***Descriptive statistics of main vital parameters on Visits 1 - 4***

| **Parameter** | **Mean** | **SD** | **SEM** | **95 % CI**  **lower** | **95 % CI upper** | **Min** | **Max** |
| --- | --- | --- | --- | --- | --- | --- | --- |
|  | ***Visit 1 (before administration)*** | | | | |  |  |
| Body temperature, °С | 36.34 | 0.27 | 0.05 | 36.24 | 36.44 | 35.8 | 36.8 |
| Blood pressure (systolic), mmHg | 118.1 | 9.25 | 1.69 | 114.65 | 121.55 | 104 | 135 |
| Blood pressure (diastolic), mmHg | 75.9 | 7.55 | 1.38 | 73.08 | 78.72 | 62 | 88 |
| Heart rate, beats per minute | 75.33 | 6.9 | 1.26 | 72.76 | 77.91 | 63 | 88 |
| Respiratory rate, numbers per minute | 14.97 | 0.81 | 0.15 | 14.66 | 15.27 | 14 | 17 |
|  | ***Visit 1 (30 min after administration)*** | | | | |  |  |
| Body temperature, °С | 36.44 | 0.2 | 0.04 | 36.37 | 36.51 | 36 | 36.7 |
| Blood pressure (systolic), mmHg | 116.43 | 9.75 | 1.78 | 112.79 | 120.07 | 93 | 134 |
| Blood pressure (diastolic), mmHg | 76.1 | 7.11 | 1.3 | 73.44 | 78.76 | 60 | 88 |
| Heart rate, beats per minute | 74.13 | 5.3 | 0.97 | 72.15 | 76.11 | 62 | 85 |
| Respiratory rate, numbers per minute | 14.8 | 0.85 | 0.15 | 14.48 | 15.12 | 14 | 17 |
|  | ***Visit 1 (60 min after administration)*** | | | | |  |  |
| Body temperature, °С | 36.49 | 0.18 | 0.03 | 36.42 | 36.55 | 36.1 | 36.7 |
| Blood pressure (systolic), mmHg | 116.6 | 9.91 | 1.81 | 112.9 | 120.3 | 95 | 138 |
| Blood pressure (diastolic), mmHg | 74.4 | 7 | 1.28 | 71.79 | 77.01 | 60 | 88 |
| Heart rate, beats per minute | 73.33 | 5.47 | 1 | 71.29 | 75.37 | 66 | 87 |
| Respiratory rate, numbers per minute | 14.8 | 0.81 | 0.15 | 14.5 | 15.1 | 14 | 17 |
|  | ***Visit 1 (2 h after administration)*** | | | | |  |  |
| Body temperature, °С | 36.44 | 0.2 | 0.04 | 36.37 | 36.52 | 36 | 36.7 |
| Blood pressure (systolic), mmHg | 117.4 | 9.36 | 1.71 | 113.9 | 120.9 | 101 | 134 |
| Blood pressure (diastolic), mmHg | 76 | 6.24 | 1.14 | 73.67 | 78.33 | 63 | 88 |
| Heart rate, beats per minute | 73.43 | 5.96 | 1.09 | 71.21 | 75.66 | 65 | 89 |
| Respiratory rate, numbers per minute | 14.9 | 0.92 | 0.17 | 14.56 | 15.24 | 14 | 17 |
|  |  |  | ***Visit 2*** |  |  |  |  |
| Body temperature, °С | 36.38 | 0.22 | 0.04 | 36.3 | 36.46 | 36 | 36.7 |
| Blood pressure (systolic), mmHg | 116.9 | 9.6 | 1.75 | 113.31 | 120.49 | 102 | 133 |
| Blood pressure (diastolic), mmHg | 76.1 | 6.1 | 1.11 | 73.82 | 78.38 | 65 | 85 |
| Heart rate, beats per minute | 74.17 | 5.5 | 1 | 72.11 | 76.22 | 64 | 87 |
| Respiratory rate, numbers per minute | 14.87 | 0.78 | 0.14 | 14.58 | 15.16 | 14 | 17 |
|  |  |  | ***Visit 3*** |  |  |  |  |
| Body temperature, °С | 36.35 | 0.25 | 0.05 | 36.25 | 36.44 | 35.8 | 36.8 |
| Blood pressure (systolic), mmHg | 118.43 | 9.28 | 1.69 | 114.97 | 121.9 | 101 | 136 |
| Blood pressure (diastolic), mmHg | 75.3 | 5.61 | 1.02 | 73.21 | 77.39 | 65 | 87 |
| Heart rate, beats per minute | 72.1 | 5.47 | 1 | 70.06 | 74.14 | 64 | 85 |
| Respiratory rate, numbers per minute | 15 | 0.87 | 0.16 | 14.67 | 15.33 | 14 | 17 |
|  |  |  | ***Visit 4*** |  |  |  |  |
| Body temperature, °С | 36.4 | 0.19 | 0.03 | 36.33 | 36.47 | 36.1 | 36.7 |
| Blood pressure (systolic), mmHg | 116.27 | 7.8 | 1.42 | 113.35 | 119.18 | 103 | 131 |
| Blood pressure (diastolic), mmHg | 75.2 | 6.28 | 1.15 | 72.85 | 77.55 | 65 | 88 |
| Heart rate, beats per minute | 73.13 | 5.75 | 1.05 | 70.99 | 75.28 | 62 | 83 |
| Respiratory rate, numbers per minute | 14.87 | 0.73 | 0.13 | 14.59 | 15.14 | 14 | 16 |
